# The Effect of Surface Topography Feature Size Density and Distribution on the Results of a Data Processing and Parameters Calculation with a Comparison of Regular Methods

**DOI:** 10.3390/ma14154077

**Published:** 2021-07-22

**Authors:** Przemysław Podulka

**Affiliations:** Faculty of Mechanical Engineering and Aeronautics, Rzeszow University of Technology, Powstancow Warszawy 8 Street, 35-959 Rzeszów, Poland; p.podulka@prz.edu.pl; Tel.: +48-17-743-2537

**Keywords:** surface topography, areal form removal, noise suppressions, end-effect, surface topography features, valleys, dimples, oil pockets

## Abstract

In this paper, the influence of occurrence of surface texture features on the values of surface topography parameters calculated after the application of various data processing techniques was presented. Different types of surface topographies were considered, as follows: cylinder liners, some with additionally burnished dimples, turned, ground, milled, laser-textured, composite, ceramic, or isotropic in general. The effects of feature size on the areal form removal, noise suppressions, or end-effect reducing in surface texture measurements were studied. The variations of the ISO 25178 standard surface topography parameters were taken into consideration in detail. It was assumed that some of the feature sizes, distributions, and densities have a substantial impact on the values of surface topography parameters calculated after applications of regular (commonly used) algorithms and procedures, defined as basic operations, provided for raw surface texture data obtained directly from the measurement process. In the end, some of the practical applications for receiving the relevant values of surface topography parameters were proposed.

## 1. Introduction

The surface topography (ST) is created in the last stages of the manufacturing process. The results of its analysis, including calculating the values of ST parameters, can affect the classification of the produced parts as those suitable for efficient operation. In fact, the ST is often called a fingerprint of the manufacturing process that many valuable and accurate information, e.g., wear resistance [1,2], lubricant retention [3], sealing [4], friction [5], fatigue [6], material contact in general, can be gained directly from its studies. Moreover, ST analysis is often an integral part of process control.

Even the precise method (technique) of ST measurement may not produce accurate results when the processes of data analysis are not provided in a conscious way. Necessary ST parameters for evaluating the tribological behavior of ‘engineering surfaces’ [7] are calculated after form removal [8]. Processes of shape and waviness separation have been studied in many research papers that the improper selection of reference planes may affect the values of surface texture parameters and the accuracy of detail applications. Very popular in the process of areal form removal are polynomials. It was found that oil pockets occurrence can be caused by a significant inaccuracy in the selection of reference planes by the polynomials [9]. Polynomials of various degrees are commonly used (available in the commercial software) algorithms for the separation of form in surface topography measurements [10]. Very often, the process of areal form removal is performed, the distortions of some of the features from surface texture are received, such as dimple (valley) flatness [11], when the degree of polynomials is too high. On the other hand, the form cannot be entirely removed from the raw measured data as the too-small degree is applied. The valley distortion can radically affect the process of calculation of ST parameters that incorrect value determination can cause classification of properly made parts as a lack and its rejection.

In previous studies, the effect of valley depth was considered [12] for the process of areal form removal by the polynomials or regular, widely used in the characterization of engineering surfaces, Gaussian filters, e.g., regular Gaussian [13], regression [14] or robust [15] approaches. A filter with the transmission characteristic described by the Gaussian curve is the most common filter used in surface metrology to separate roughness, waviness, and form [16]. Gaussian reference line, namely a profile line obtained by Gaussian low-pass filtering, is legalized as the reference line for surface roughness evaluation [17]. Therefore many newly proposed procedures are often compared with results received by the Gaussian filtering techniques. Regardless of many papers considering areal form removal of engineering surfaces, there still is no adequate explanation of the influence of selected features sizes, distributions, and densities on the results of separation of roughness, waviness, and form.

In general, received from the ST measurement process, a signal is composed of the background signal characterized by low frequency, a noise signal of high-frequency, and finally by the useful signal [18]. Those signals need to be processed separately. The high-frequency noise can greatly affect the stability of slope estimation in ST analysis. Generally, this type of noise can be caused by instability of the mechanics with any influences from the environment or by internal electrical noise. In most cases, the high-frequency noise is the result of vibration [19]. The effect of environmental noise, in general, can be reduced with wavelength scanning interferometry [20]. A filter that removes small-scale lateral components, e.g., high-frequency signal, from the surface is defined as an S-filter, and, simultaneously, the filter that eliminates the form from the surface is described as an F-operator. The surface obtained by the above two operations is specified as an S-F surface [21]. The results of the application of both operations (filters) can be directly affected by the characterization of the features in the analyzed detail. Removing the S-components of the obtained measurement signal, defined as a high-frequency noise, is one of the poorly researched issues in surface metrology. The high-frequency errors can also be caused by the electrical noise in the sensor output, irrespective that nowadays, the use of sensors is more robust to environmental issues [22]. Reduction in this type of error can provide a better resolution, which also reduces the bandwidth of the sensor. However, there are no proposals for the reduction in high-frequency noise with no bandwidth modifications.

In White Light Interference (WLI), where the non-uniform distribution of the light intensity occurs, the extracted signal is liable to be skewed and asymmetric and to contain a lot of high-frequency noise. The noise can increase with bandwidth; therefore, reduction in the bandwidth can reduce the selected amount of high-frequency noise [23]. Application of the wavelet transform can reduce different frequency components of the interference signal [24]. The signal can be separated into a non-overlapping band, which is an advantage in the noise reduction and the signal de-noising process [25]. Moreover, wavelet transform combined with soft threshold filtering and homogenization in the phase evolution algorithm can eliminate the distortion of the interference signal as well [26]. Generally, wavelet transform can be highly advantageous for surface roughness evaluation, texture extraction, and S-F surface definition [27].

Considering all the above, there is no appropriate response on the influence of the size, distribution (location), and density of selected (e.g., dimples, oil pockets, scratches, valleys in general) features on the values of ST parameters calculated after data processing operations, such as form, waviness, and high-frequency noise separations. Moreover, the more complicated the manufacturing process, the more adequate proposal of algorithm application is required. From all of the currently existed and commonly applied algorithms, procedures, schemes, and approaches, thoroughly described in many scientific articles, there is a high bias error when all those methods are used. From that point of view, the validation of the commonly used (available in the commercial software) algorithms and procedures seem to be an important issue that errors received during the data is processed may not allow providing adequate results. More sophisticated procedures may not ensure receipt of correct data. Consequently, in this paper, the effect of surface topography features on the ISO 25178 parameters calculation after S-filtration and F-filtration processes were taken into sufficient consideration. For extraction of the S-components and F-components from the raw measured data, regular (commonly used) algorithms are presented and compared, respectively. Moreover, results are presented for both the areal (2D) and profile (3D) characterizations.

## 2. Materials and Methods

### 2.1. Measurement Process

The studied details were measured by various techniques, stylus or optical. Some of the measurement was provided by the stylus instrument Talyscan 150 with a nominal tip radius of about 2 μm, height resolution about 10 nm, and the measured area 5 by 5 mm (1000 × 1000 measured points). The sampling interval was 5 µm. The measurement speed was 0.5 or 1 mm/s. Its influence was not the preliminary of this research that it was studied in previous items.

The non-contact measurement was completed with the white light interferometer Talysurf CCI Lite, the height resolution 0.01 nm, the measured area 3.35 by 3.35 mm with 1024 × 1024 measured points, the spacing 3.27 µm. The influence of the sampling on the values of areal texture parameters was not studied in this paper. In both cases, the measurement was repeated 3 times, and the mean values were considered.

The following ST parameters, from the ISO 25178 standard, were measured and analyzed: root mean square height *Sq*, skewness *Ssk*, kurtosis *Sku*, maximum peak height *Sp*, maximum valley depth *Sv*, the maximum height of surface *Sz*, arithmetic mean height *Sa*, autocorrelation length *Sal*, texture parameter *Str*, texture direction *Std*, root mean square gradient *Sdq*, developed interfacial areal ratio *Sdr*, peak density *Spd*, arithmetic mean peak curvature *Spc*, core roughness depth *Sk*, reduced summit height *Spk*, reduced valley depth *Svk*, surface bearing index *Sbi*, core fluid retention index *Sci,* and valley fluid retention index *Svi*.

### 2.2. Analyzed Surfaces

**Figure 1:**
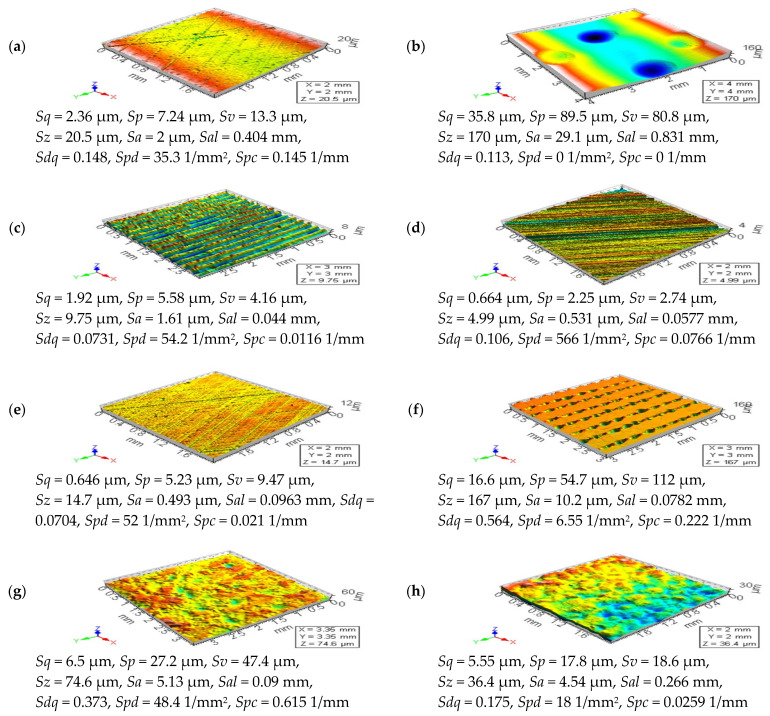
Examples of analyzed surfaces with selected surface topography parameters correspondingly, as follows: cylinder liner (**a**) with additionally burnished dimples (**b**), turned (**c**), ground (**d**), milled (**e**), laser-textured (**f**), composite (**g**), and ceramic (**h**) topographies.

To confirm the suitability of the analyzed, commonly used procedures (approaches and filters), various types of surface topographies were taken into consideration. More than 10 surfaces (modeled and measured in particular) were studied, but only some of them, consistent with general observations to all analyzed data, are presented in detail. In Figure 1, examples of each type of analyzed detail (surfaces) are presented with an isometric view and selected surface topography parameters. The first type of surface is the plateau-honed cylinder liners (a), some with additionally burnished oil pockets (b). Cylinder liner texture can be presented as a representative example of functional surfaces. The progress from surface engineering to the functional surface was one of the main objectives in many types of research [28]. Liners have surface topographies with a cross-hatch pattern generated in a finishing process known as honing, which is an abrasive machining process in which material is cut away from the workpiece using abrasive grains that are bound together with an adhesive to form a honing stone [29]. A plateau-honed cylinder surface, generated in a finishing process, establish simultaneously both the sliding properties of a smooth surface and a great ability to maintain oil on a porous surface; therefore, it is believed that a plateau-honed surface improves the lubrication and reduces friction and wear [30]. Consequently, multi-process topographies, which bear traces of two or more processes, are becoming increasingly important from a functional point of view. A typical example of the textured surface is the plateau-honed cylinder liner surfaces with networks of micro reservoirs, also correctly named as dimple, cavities, or, simply, oil pockets. Previous studies prove that this type of surface texture has great sliding properties and an outstanding ability to preserve the oil in its rough topography. Analysis of both types of surface topographies, plateau-honed cylinder liners, some after the process of oil pockets burnishing, may be particularly significant in the tribological point of view. Other types of tribologically important details are those after the turning (c) or grinding (d) processes. Turned piston skirts work with plateau-honed cylinder liners. It was found that the waviness layout is largely disrupted when surfaces contain curvature and, consequently, imperfections in the manufacturing process cause many of such disruptions [31]. Analysis of the turned surfaces can be performed when the topographies of brake pads are thoroughly evaluated [32]. Analysis of the milled textures (e) is especially crucial in the last stage (end) of the milling process. Milling is a widely employed material removal process for different materials that are characterized by high material removal rate and, generally, machining leads to high friction between tool and workpiece that can result in high temperatures, impairing the dimensional accuracy and the surface quality of products [33].

Laser texturing is one of the machining methods used to prepare a workpiece surface with various microstructures. The tribological behavior [34] of the contact surface can be affected by surface roughness if the liquid film is formed to have a thickness with the same order of magnitude [35] and, simultaneously, that can be affected by the texturing of the surface topography. Selected STs produced with laser melting methods (SLM), which improves the surface quality [36], can be classified within a group of complex textures. They usually feature significant topographic details in multiple scales, with a mixture of high and low aspect-ratio formations, high slopes, undercuts and deep recesses, etc. Therefore these types of surface topographies (f) are challenging to measure or analyze, e.g., the small cavities may become protrusions, while regular hemispheric shapes (e.g., spatter particles) may seem to be irregular.

Generally different from the metal surface after machining is the engineering, isotropic composite (g), or ceramic surfaces (h). The surface quality of workpiece during ceramic grinding is an ever-increasing concern in industries nowadays that the importance of the surface finish of a product depends upon its functional requirements. Since the surface finish is governed by many factors, its experimental determination is laborious (time-consuming), so the establishment of a model for the reliable prediction of surface roughness is still a key issue for ceramic grinding [37]. Measurement and analysis of this type of ground surface seem to be still a mammoth task to be solved.

### 2.3. Applied Methods

#### 2.3.1. Procedures for an Areal form Removal

Algorithms and procedures proposed in the paper can be roughly divided into two groups, based on the issue taken to solve. The first group of approaches implemented was those for the separation of the form from the results of surface topography measurements. Areal form removal in surface metrology can be provided with plenty of Gaussian filters. Moreover, the Gaussian filters were proposed according to the ISO 16610-21 [38], providing the official standard defining the Gaussian filter for open or closed profiles. For surfaces, the equivalent definition is given in ISO 16610-61 [39]. A robust Gaussian regression filter, defined in ISO 16610-31, has its mean plane (line) correctly following the general trend of the surface (profile) without being disaggregated by outliers, outlier-like errors, or other extraordinary values. The robust Gaussian filter is an iterative algorithm that calculates local weights based on the distance between the primary profile and the waviness profile [40]. All the commonly used Gaussian filters proposed, e.g., in the commercial software, can allow receiving relevant results in the surface topography filtering process that make the analysis of the results obtained more qualitative.

Contrary to the Gaussian filtering techniques, the least-square fitted polynomial plane of a different (*n*-th) order is proposed in plenty of software appliances. This type of data processing method is more robust from the end-effect than the regular Gaussian algorithms. Nevertheless, it was found in the previous studies that the valley size has a huge impact on the areal form removal. Generally, when the surface contained the dimples, the form removal by the commonly used procedures, e.g., least-square fitted polynomial plane of *n*-th order or regular Gaussian filters, was not provided. To resolve the problem of valley sizes (depth, width), the valley excluding method (VEM) [41], based on the extraction and fulfilling of valleys, was suggested. This technique provided better results, especially when the valleys (oil pockets) were edge-located.

Regardless of the above statement, the effect of feature (e.g., oil pockets, dimples, scratches, valleys in general) distribution, its density and sizes (especially widths) on the results of areal form removal by the regular, often-used algorithms was not comprehensively studied and, simultaneously, only a few hints appeared in this topic.

#### 2.3.2. Algorithms and Approaches for Characterizations of the High-Frequency Errors

Generally, the process of suppression of the high-frequency noise can be effectively separated into two different, albeit dependent, processes such as definition (detection) and removal (extraction and reduction). Therefore, the procedures for the high-frequency measurement errors characterizations were classified into those applied for detection of the S-components of the received measurement signal and, correspondingly, those with the suppression performance.

##### Approaches Considered for the Detection of the High-Frequency Noise

**Figure 2:**
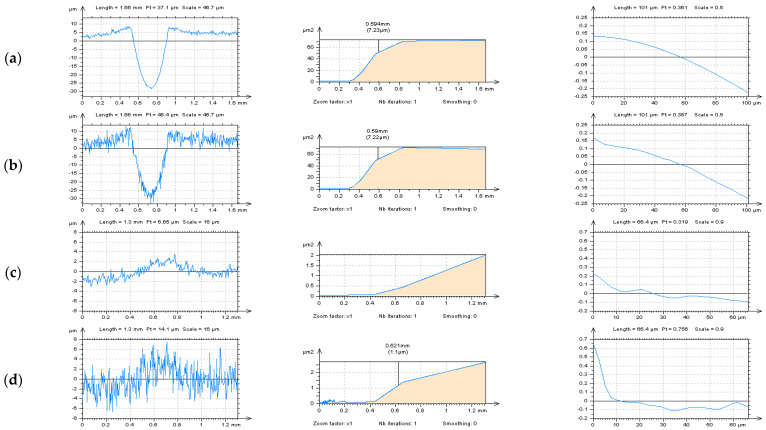
Profiles (left column), their PSDs (middle), and ACFs (right column), respectively, extracted from the turned cylindrical surface containing dimples by the horizontal (**a**,**b**) and treatment trace (**c**,**d**) technique.

The detection of high-frequency noise can be provided considering various techniques. In general, the high-frequency components of the measurement signal should be visible on the Power Spectral Density (PSD) graphs that the PSD describes how the power of a signal or time series is distributed over the different frequencies [42]. The PSD, in its two-dimensional form, has been designated as the preferred means of specifying the surface roughness on the draft international drawing standard for surface texture [43] and can be freely applied in many surface studies. For example, the surface quality can be favorably compared using PSD, e.g., for dry and Minimum Quantity Cooling Lubrication (MQCL) turning process [44]. Furthermore, the PSD technique provides the average frequency spectrum of the acquired signals, making possible the determination of the actual contribution of the tool wear mechanisms. Finally, the average PSD, which is the area of the PSD signal curve that gives the energy of the signal, can be obtained. Therefore, a methodology for the detection of wear mechanisms and determination of the end of life of the cutting tool based on the acoustic emission signals was proposed using PSD. Based on the definition of the PSD technique, which can be described as the estimation of the distribution of the total power of the signal in the frequency domain from a finite recording of a sequence of stationary data [45], the detection of some of the frequency errors, e.g., those in the high-frequency domain, can be proceeded with the PSD appliances. When the high-frequency noise was detected, it was found that the profile (2D) PSD analysis might be more convincing than the areal (3D) studies [46]. Examples of PSD detection procedures of high-frequency errors from the raw surface texture measurement data of turned details are presented in Figure 2.

The type of profile extraction technique was fitted to the type of surface studied. For turned cylindrical surfaces containing deep and wide dimples, the out-of-feature approach was applied. This method is based on the analysis of this part of the surface where the traces of manufacturing treatment, e.g., dimples, scratches, oil pockets, valleys in general, do not occur. The application of this technique provided better noise recognition by the PSD analysis. The detection process of high-frequency errors with autocorrelation function (ACF) characterization was useful regardless of the out-of-feature or treatment trace schemes.

Specification of the S-F surface can also be provided by the analysis of the results extracted from the raw measured data. For example, to determine the suitability of the F-operator algorithm, F-filter, or F-procedure in particular, the specification of the form surface (F-surface) and the short-wavelength noise surface (S-surface) [47] can become exceedingly valuable. Simplifying, analysis of the F-surface and the S-surface can be particularly significant for the selection of the procedures for removing irrelevant features, such as form (shape and waviness) or measurement noise in selected bandwidth, from the raw measured surface topography data.

##### Filters Applied for the Reduction in the Influence of High-Frequency Errors

There were many filters proposed for the removal of the measurement noise from the results of surface topography measurements. As it was mentioned before, surface metrology is often supported by plenty of Gaussian filtering algorithms. A denoising Gaussian S-filter [48] (DGS-F) is an example of S-filters for the removal of short components from the received measurement results (signal). Spatial oriented Gaussian filter was applied previously with convolution-based fringe pattern denoising method [49], the proposal has an outstanding performance against noise. Moreover, the Gaussian filter is ideally suited for smoothing surfaces with rich features. Both S-F surface and waviness surface can be obtained from a single filtering procedure without phase distortion in either of the separated components [50].

Generally, noise, especially in the high-frequency domain, can be effectively suppressed by the various averaging filters. Typical examples of this type of filter are denoising moving average (DMAF) and denoising median (DMF) algorithms. The DMAF is the easiest digital filter to understand and use. It is the most common filter in the analysis of a signal. In spite of its simplicity, the DMAF approach is optimal for a common task such as reducing noise [51]. In turn, it was found that the ranges of effective spatial frequency could be extended through DMF without destroying the properties of the fractal surface. The results of this research were particularly important for characterizing imaging optical systems accurately that the median filtering expands the effective spatial frequency, enhances the effective resolution, and significantly increases the use of the optical profiler without destroying the properties of the fractal surface [52].

Increasingly popular in the characterization of surface topography are wavelets. Wavelet transforms can divide functions into different scale-frequency components, and then each component can be studied with a resolution matched to its scale [53]. One of the classifications can divide wavelets into orthogonal [54] and biorthogonal [55]. Progress in wavelet analysis lets use orthogonal wavelets to decompose turned, milled, and ground surfaces, as well as evaluate tool marks, machining vibrations, and machine-tool errors [56]. The biorthogonal wavelet transform is very suitable for isotropic surfaces but has difficulty with surface topography with various scale scratches such as those in plateau-honed surfaces or worn biomedical surfaces. It is caused that in a discrete biorthogonal wavelet transform, small shifts in the position of the surface can result in a completely different distribution of ‘energy’ among the wavelet components. To extract and reconstruct a surface topography with various scales scratches, such as those in plateau-honed surfaces and worn biomedical surfaces, a complex wavelet model should be proposed [57]. Wavelet transforms can decompose a signal into several scales that represent different frequency bands, and at each scale, the position of the signal’s instantaneous structures can be determined approximately. Such a property can be used for the denoising process [58]. According to the versatility of the wavelet applications, the wavelet denoising filter (DWF), based on the orthogonal Daubechies wavelet [59], was proposed.

All of the above filters, except the wavelet approaches, are available in the commercial software that the main purpose of their application was to determine its usage for the process of reduction in the high-frequency errors from the results of surface texture measurements.

## 3. Results and Discussion

### 3.1. Areal form Removal

#### 3.1.1. The Effect of Feature Occurrence on the Areal form Removal by Regular Procedures

**Figure 3:**
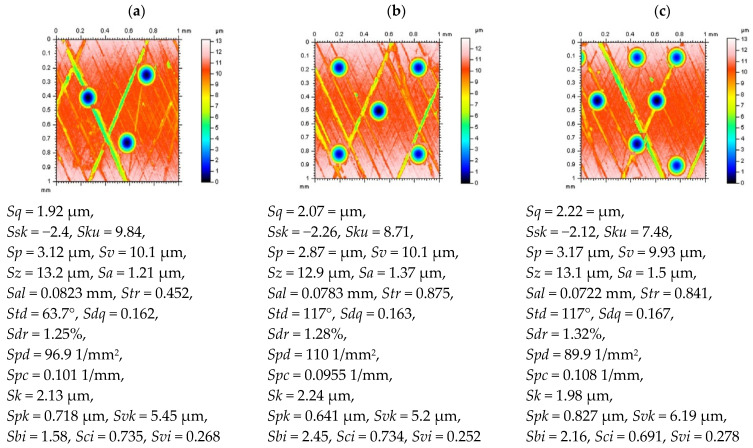
Contour map plots of details and their selected surface topography parameters, respectively, received from honed cylinder liner texture with different densities of the dimples: Surf1 with low density (**a**), Surf2 with medium density (**b**), and Surf3 with high density (**c**) of valleys.

The effect of feature (dimples, valleys, scratches) size, density, and distribution on the results of areal form removal commonly used (available in commercial, TalyMap Gold software) procedures were taken into consideration. In Figure 3, contour map plots and surface texture parameters, correspondingly, of three plateau-honed cylinder liner topographies with different densities and distribution of the dimples (oil pockets) are presented. It was found that the value of the *Sq* and *Sa* parameters increased, but the values of the *Sz* parameter changed slightly when the density of oil pockets was larger. No significant changes in the maximum height parameter value are caused by the constant depth of the ST features, oil pockets in particular. Noteworthy is that the ‘dominant’ texture direction is related to the number (and density) of the features, such as dimples (oil pockets) and (relatively) deep scratches. When the number of dimples was greater than the number of deep scratches, the *Std* value was affected by the direction of the scratches. Otherwise, the texture direction parameter value was dependent on the direction of the oil pockets (dimples). Therefore, the density of the valleys can affects the value of the *Std* parameter more than the direction of the scratches created by the honing process.

Selection of the procedure for an areal form removal of plateau-honed cylinder liner surfaces, especially those containing additionally burnished dimples, was proposed with minimizing (maximizing) of the values of *Sk* and *Spk* (*Svk*) parameters [9]. It was indicated that the core roughness of the surface should be minimized when the depth of the valleys was maximized simultaneously, the dimples could not be distorted, it was found that the oil pockets are often flattened when an improper algorithm was applied. In line with the previous research results, the 2nd degree of polynomial seemed to be the most encourages when plateau-honed cylinder liner contained oil reservoirs, the 4th degree (Poly 4th) was proposed for honed cylinder liners with no additionally created dimples. Nevertheless, the 2nd-degree least-square fitted polynomial plane (Poly 2nd) may not be appropriate when dimples were distributed unevenly (Figure 3c). The values of the *Sk* parameter were minimized when oil pockets were located equally over the entire analyzed detail. In general, the polynomial plane of the 2nd or 4th degree was more sensitive to the location (distribution) of the features (oil pockets) than their densities. Similar observations can be presented for areal form filtering by the Gaussian regression (Gauss) or robust (Robust) methods. When the distribution of valleys was equal on the entire surface, both procedures made it possible to obtain acceptable results. Nonetheless, a robust filter minimized the sum of the *Sk* and *Spk* parameters when, concurrently, the value of the *Svk* parameter increased according to the regular Gaussian regression filter application. The description of all studied parameters was presented in Table 1.

#### 3.1.2. Selection of F-Surface (Reference Plane) with Valley Analysis

**Figure 4:**
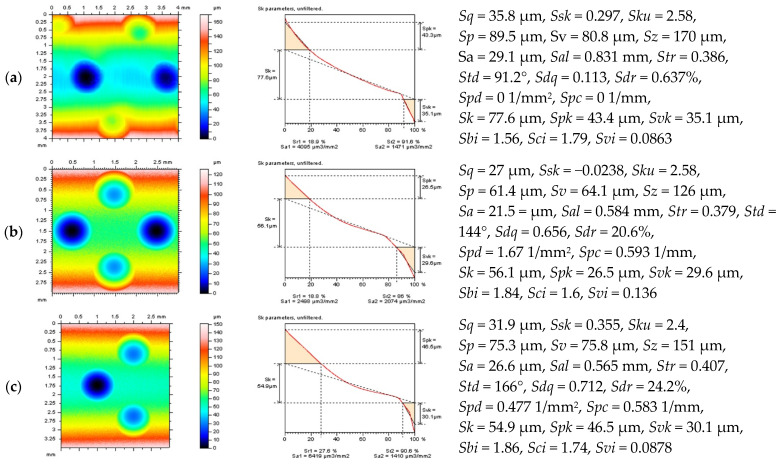
Contour map plots (left column), material ratio curves (middle), and surface texture parameters (right column) of the cylindrical surfaces after turning process (**a**) and isotropic (**b**,**c**) with different dimple distribution.

Apart from the influence of the density of surface features, the size and distribution of dimples, scratches, and valleys, in general, affect both the results of processing of the raw measured data and the values of the calculated surface topography parameters. In Figure 4, turned or isotropic cylindrical surfaces are presented, containing dimples with a different location, as follows: edge-located (a,b) with different densities where detail (b) contain the dimples located on (near to) the edge of an analyzed surface, and center-distributed (c) where valleys were quite far (more than 0.4 mm) from the edge of a considered surface.

From the analysis of the isometric views of the details, it was found that the polynomial form removal (for various degrees) was influenced if the feature (valley) was evenly situated more than the dimple size or density. For both filters, regular Gaussian regression and robust Gaussian regression, that when the distance of the dimple(s) to the edge of analyzed detail was smaller than the cut-off value of the applied filter, e.g., 0.8 mm, then the distortion (usually flatness) of the valleys increased. Moreover, when the distance between dimples was smaller than the cut-off value irrespective of the dimple-to-edge distance, the feature exaggerations also tends to increase.

It was also found in previous studies [12] that the feature depth, especially when deep dimples were considered, influenced the selection of procedure for an areal form removal. Usually, when the valley depth increased, the distortion of the F-surface also increased for least-squares fitting methods, in general.

It was found (Table 2) that the values of *Sk* and *Spk* (*Svk*) parameters were minimized (simultaneously maximized) when the least-square fitted of the 2nd-degree polynomial plane was established, the sum of values of the *Sk* and *Spk* parameters was minimized. The robust filter can be applied alternatively that the value of core roughness (*Sk*) was minimized when a robust approach was used, the regular Gaussian filter for the calculation of the parameters after form removal is not suggested that the valleys were disaggregated seriously, usually flattened, that the value of reduced valley depth (*Svk*) also decreased significantly.

When the analyzed detail did not contain edge-distributed features, dimples in this particular example, the ‘minimizing technique’, widely commented in reference [9], indicated the robust filtering as this most useful for the separation of the form from the topographies containing deep/wide features such as burnished oil reservoirs. However, the form-separation algorithm should be selected carefully that the value of the *Sv* parameter was maximized, but, unfavorably, the values of the *Sp* parameter also increased. For all of the considered feature densities, the F-surface defined by the polynomial of 2nd degree provided the most encouraging results that the sum of the heights of the core and plateau components of the surface (*Sk* and *Spk*) was minimized. The other, higher (4th) degree of the polynomial plane might be valuable when the *Sp*-*Spk* sum was calculated, but the valley depth (*Svk*, *Sv*) was reduced, which might indicate the flatness (distortion) of the oil pockets. This disaggregation of the valley capacity may result in the erroneous estimation of the surface sliding properties and caused a false classification of the manufactured parts.

From all of the above, it is suggested to select the form-separation scheme with both visual and parametric assessments that the minimization (maximization) of the *Sk*-group parameters may not be convincing when the distortion of the surface features occurs.

#### 3.1.3. Reducing the Influence of an End-Effect in the Analysis of Surface Topography

A very complicated and pressing problem is the effect of the boundary results in digital filtering. The weight modification, simply called the regression scheme, has been improved for machined surfaces [60], that the effect of boundary conditions in the digital metrology filtering was reduced. The regression modification was often proposed, considering surface texture analysis, for the Gaussian filters, especially when robust properties [61] were reasonably required. Robust and regression qualities of the filter improved the functionality of the algorithms applied; nevertheless, distortions of some of the surface texture features still occurred when they were located in (near) the edge areas of the analyzed detail.

**Figure 5:**
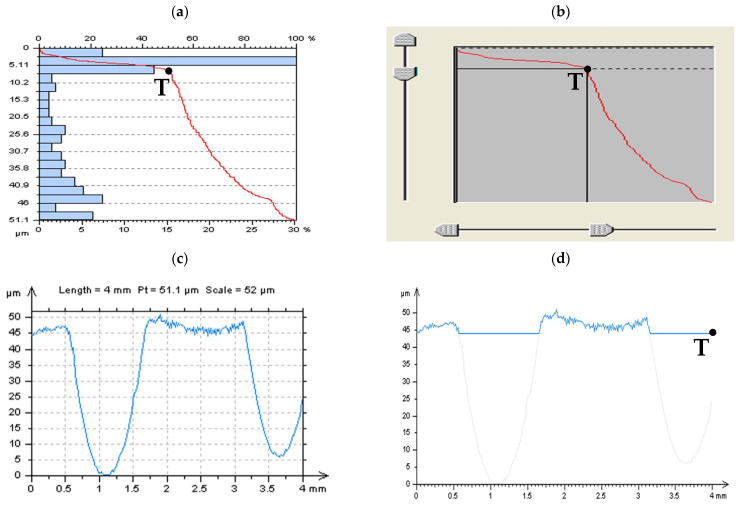
An Abbott-Firestone curve (**a**), thresholding (value) point T (**b**), measured profile (**c**), and the same profile with filled features/valleys (**d**) extracted from the cylindrical surface containing dimples.

Contrary to the robust and regression algorithms, the procedures based on the feature extraction, filling, and omitting, in particular, may provide reasonable results for areal shape and waviness separations. This technique was suggested for cylindrical surfaces containing deep or wide features [41] when their width is greater than half of the F-filter cut-off value. The method of selection of the thresholding value (b) was presented in Figure 5 with Abbott-Firestone (a) and profile characterizations (c,d) by indicating the T point (value). Excluded in the proposed technique, valleys did not influence the accuracy of the areal form removal process provided by regular algorithms (filter or a least-square fitted polynomial planes). The distortion of both features (valleys) and edges of an analyzed surface topography detail was reduced. For validation of the proposed procedure analysis based on the characterization of the surface topography parameters, profiles (2D) and specified areas (3D) of studied details were taken into consideration.

**Figure 6:**
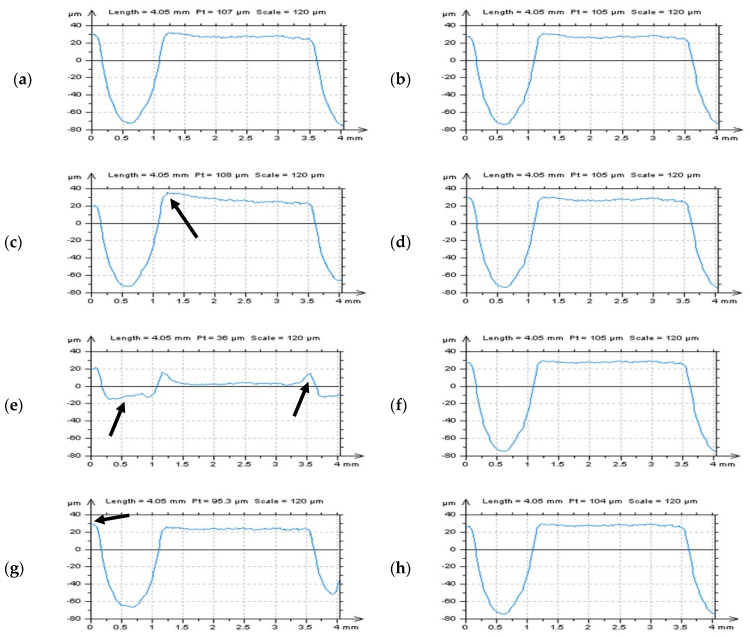
Profiles extracted from the turned cylindrical surface containing deep and wide dimples after an areal form removal by the polynomial of 2nd (**a**,**b**) or 4th (**c**,**d**) degree, application of the commonly used regression (**e**,**f**), or robust (**g**,**h**) Gaussian filter, cut-off = 0.8 mm, regular algorithms (left column) and modified procedures with a thresholding method (right column).

In Figure 6, examples of profiles received from a surface after reducing the end-effect in the process of an areal form removal are presented. In the left column, the details after the application of regular methods (F-surfaces created by the polynomials or Gaussian filters) are presented. When dimples were located near (on) the edge of an analyzed surface, the distortion of both oil reservoirs and edge areas increased enormously. Oil pockets were distorted (flattened), especially when a widely used regular Gaussian filter (cut-off = 0.8 mm) was applied. Application of the thresholding method (right column) allowed to reduce the effect of an edge form removal and minimization of possibility of a false classification of properly manufactured parts as a lack. Some of the exaggerated profile features were indicated by the arrows.

**Figure 7:**
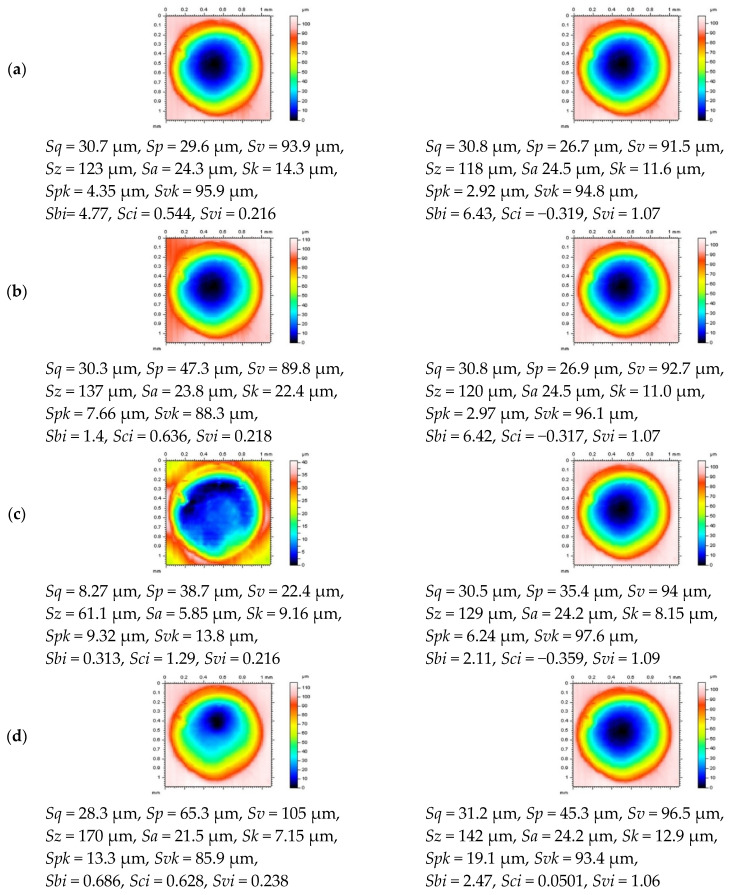
The edge-located dimple areas and surface topography parameters, respectively, received from the turned cylindrical detail containing burnished dimples, after areal form removal by the regular algorithms (left column) and the same procedures with threshold modification (right column) and after application of a polynomial plane of 2nd (**a**) and 4th (**b**) degree or regular (**c**) and robust (**d**) Gaussian regression filters with cut-off = 0.8 mm.

In Figure 7, examples of dimple details distributed in the edge-area of the analyzed surface with parameters are presented. From the analysis of contour map plots of the edge-located dimples, it was found that the application of a thresholding method allowed to reduce the distortion of the oil pockets. Usually, the values of *Sk* and *Spk* (*Svk*) parameters were reduced (increased) when the polynomial (of 2nd or 4th degree) or regular Gaussian plane was calculated. When the robust Gaussian filter was used, the application of a thresholding procedure caused an increase in the *Sk* and *Spk,* but the usefulness of a proposed method was preserved that the regular algorithms may cause the flatness of the deep and wide features, oil pockets, valleys, and scratches, in particular.

### 3.2. Analysis of the High-Frequency Measurement Errors

#### 3.2.1. The Influence of Feature Size, Density, and Distribution on the Processes of Detection of High-Frequency Errors

It was found in previous studies that the profile (2D) analysis might have been more valuable in the detection of the high-frequency components of textured surfaces than the areal (3D) assessments [46]. Thus, the detection of the high-frequency errors with an analysis of the PSD and ACF graphs was applied. It was assumed that the feature occurrence and simultaneously density affect the PSD and ACF profile noise detection.

**Figure 8:**
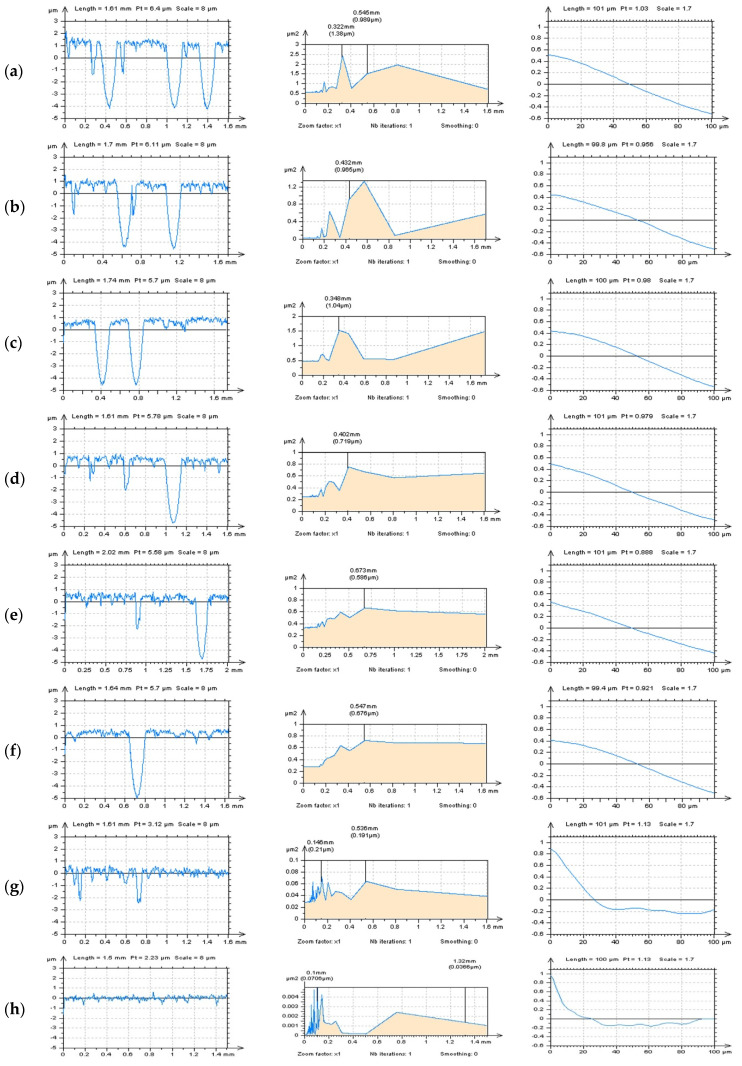
Profiles (left column), their PSDs (center) and ACFs (right column), received from stylus measured, the plateau-honed cylinder liner surface with the following number of features: three dimples (**a**), two dimples and one scratch (**b**), two dimples (**c**), one dimple and two scratches (**d**), one dimple and one scratch (**e**), one dimple and no scratches (f), two scratches but no dimples (**g**) and no dimples and no scratches (**h**) profile.

In Figure 8, profiles received from the plateau-honed cylinder liner surface, with their PSDs and ACFs, respectively, and with a various number (density) of the features (dimples, valleys, scratches) are presented, as follows: three dimples (a), two dimples and one scratch (b), two dimples (c), one dimple and two scratches (d), one dimple and one scratch (e), one dimple and no scratches (f), two scratches but no dimples (g) and no dimples and no scratches (h) profile. When profiles contained the dimples (oil reservoirs), the high-frequency components were not visible on the PSD graph (a–e in Figure 8). In this case, the number (density) of the dimples was negligible, that they did not affect the accuracy of noise detection. Even one dimple considerably reduced the possibility of high-frequency noise identification.

Moreover, the occurrence of the other features, e.g., valleys, scratches, has a negligible impact on the accuracy of the detection process. Notwithstanding, the number (density) of the scratches had a considerable influence on the disclosure of the high-frequency components when oil reservoirs did not occur (g, h). The smallest density (number) of features (oil pockets, valleys, scratches) was found the biggest applicability of the PSD method was observed. Consequently, it is thoroughly recommended to detect the high-frequency errors from the results of profile measurements of plateau-honed surface topography, considering the PSDs and ACFs applications, with no (deep, wide) feature occurrence.

**Figure 9:**
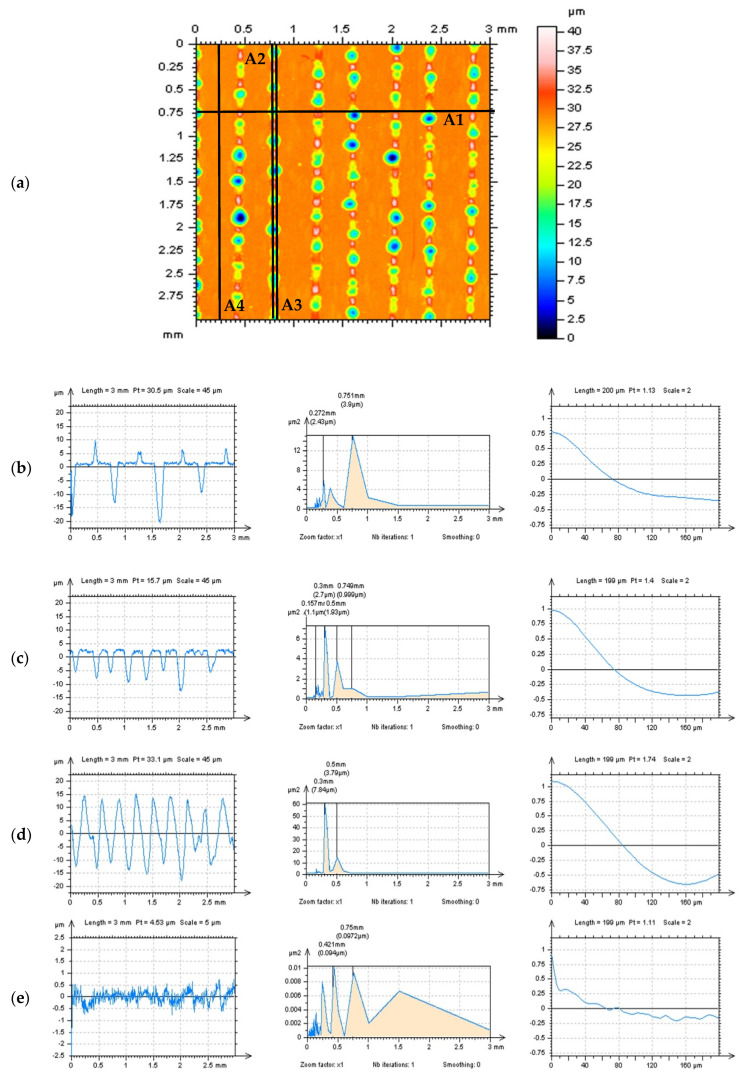
Methods of detection of the high-frequency errors by the analysis of the profiles, their PSDs and ACFs, correspondingly, extracted from laser-textured surface topography, received by extraction in different directions and areas (**a**) of analyzed detail, as follows: A1 (**b**), A2 (**c**), A3 (**d**), and A4 (**e**).

For surfaces with traces of the laser treatments (Figure 9), it was observed that the flat (with no laser-manufacturing areas) profiles can be quite valuable in the high-frequency errors detection (e) that both valleys (c) and hills (d) negatively affected the accuracy of the PSDs and ACFs noise detection with profile explorations. The shape of the center part of the ACF, in the areas of the maximum value of this function, has also been modified when the noise was observed with no-trace profiles. It was noticed that the noise profile contain the ACF with a higher degree of increase when the maximum (near the ‘1’) value was considered. This dependence was followed for each type of surface.

**Figure 10:**
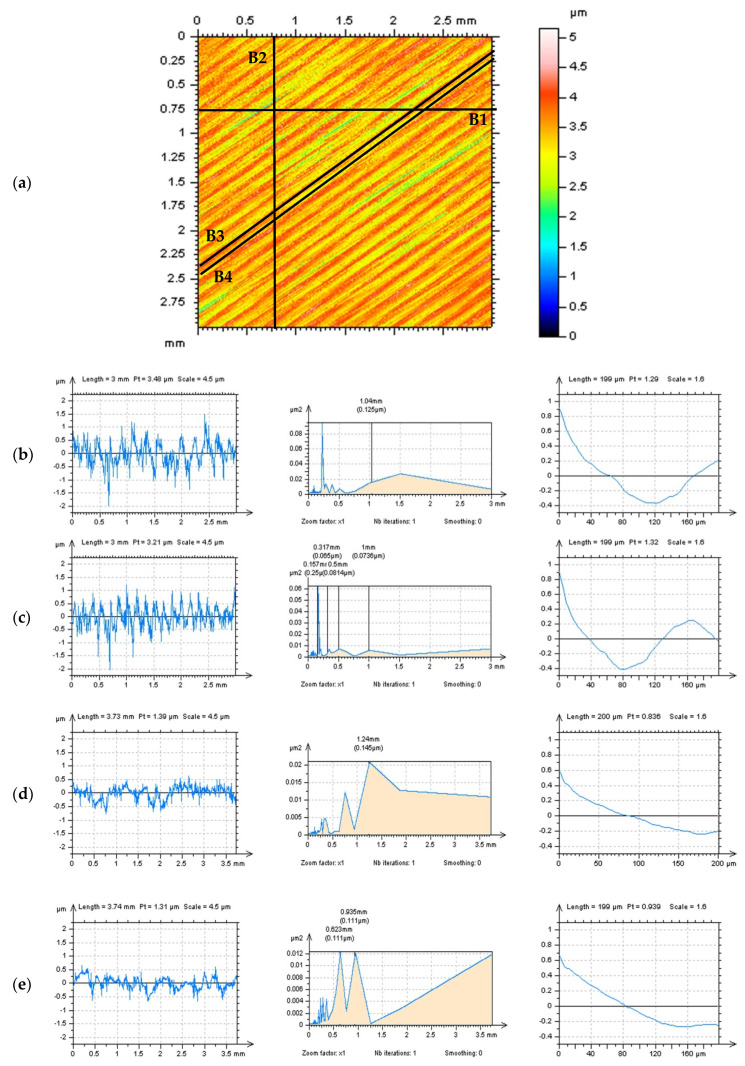
Detection of the high-frequency noise by the analysis of the profiles, their PSDs and ACFs, respectively, obtained from a milled surface with the extraction of profiles proposed in different directions (**a**): B1 (**b**), B2 (**c**), B3 (**d**), and B4 (**e**).

The ‘no-trace’ technique may be partially modified for milled surfaces (Figure 10). It was observed that horizontal (b) or vertical (c) extraction of profiles did not provide valuable information about the high-frequency noise concurrence with PSDs or ACFs graphs assessment. For this type of texture, where one direction of traces received with the manufacturing process can be defined, the direction of profile extraction for noise detection should be purposed with a direction of the traces of the surface finishing. This method can be designated (named) as a ‘treatment-(traces) direction method’ (TDM). However, in the case of the milled or turned surfaces, the TDM can be applied for the top (d) or bottom (e) area of the studied detail. It was noticed that the analysis of a ‘bottom’ profile (B4) could be much more useful in a high-frequency noise detection than the ‘top’ profile (B3) regardless of the distance of the profiles was relatively small.

Generally, it was observed that the PSD and ACF high-frequency noise profile detection is even more beneficial when the amplitude of the analyzed profile is small, does not contain the dimples, valleys, scratches, or, simultaneously, are defined with the direction of the manufacturing (finishing) process. Both techniques can be used concurrently or alternately when the PSD or ACF profile detection of the high-frequency noise is not conclusive.

#### 3.2.2. Reduction in the High-Frequency Errors with a Modeled Data Analysis

For validation of the commonly used, i.a. available in the commercial software, procedures, characterization based on the modeled data (noise) was proposed. For the data of various surface textures (start data), the high-frequency noise data were added (noise data, data with noise, or simply noise) and then removed by different filters, described in one of the previous sections. The selection of the cut-off value for noise suppressions was studied previously [47] and is also presented in the next chapter, which is dedicated to the analysis of a measured surface. It is obvious that results received after different filtering, regardless they all are dedicated to the reduction in the high-frequency components from the data, is also different [62]. Nevertheless, applying filters dedicated to the suppression of the high-frequency components of the data seems to be a logical solution.

Generally, results obtained after noise removal should be similar to those before adding noise data and, simultaneously, differences in values of the surface texture parameters should be minimized as well. Moreover, it was also concluded that the noise added to the surface should also be close to the data removed after the application of the noise-removal algorithm. Therefore both comparisons might be taken into consideration while selecting the procedures for suppressions of the high-frequency noise from measured data such as commonly used and available in regular commercial software.

For surfaces of cylinder liners after the plateau-honing process, distortion of height (amplitude) parameters was minimized when Gaussian or moving average filters were applied. Usually, differences for special parameters (*Sal*, *Str*, *Std*) were under 5% or were negligible, and when hybrid parameters were considered, the differences for root mean square gradient (*Sdq*) were slight. Nonetheless, all of those four parameters were defined previously as robust for the presence of the (high-frequency) noise [18]. Parameters related to the characterization of the peaks (*Spd*, *Spc*) underwent the greatest changes. Results after the application of various filters were also significant. Results also varied after various filtering methods for the *Sk*-group and functional indices.

When plateau-honed cylinder liner surfaces contained additionally burnished dimples, oil pockets, or other deep/wide valleys, the less distorted parameters were received when wavelet (WDF) was applied. The WDF caused the minimization of exaggerations of amplitude parameters, excluding the root mean square high (*Sq*) of the surface. For this type of surface, it was found that different filters minimized distortion of parameters from a different group, that median approach (DMF) minimized changes of *Sk* and *Spk* parameters, Gaussian denoising filter (DGS-F) reduced the most errors in computing the functional indices (*Sbi*, *Sci*, *Svi*), moving average scheme (MADF) minimized variation of the peak-dependent indicators (*Spd* and *Spc*). Distortion of most parameters was minimized by the application of the WDF algorithm; nevertheless, secondary in terms of numbers and peak density subjections, the MADF may be suggested alternatively. All of the analyses of cylinder liner surface texture parameters are presented in Table 3.

Moving average and median filters seems to be suitable for high-frequency errors reduction when turned or ground surfaces are studied. From all four compared filters, for minimization of the high-frequency errors in the measurement of milled surfaces, it is extremely difficult to select an appropriate method; nonetheless, errors in the calculation of parameters of the laser-textured surfaces are considerable reduced with an application of a DGF-S method. A method based on the Gaussian function can also be valuable when ceramic, composite, isotropic in general, surfaces are studied. All of the values of the mentioned parameters are presented in Table 4 and Table 5.

#### 3.2.3. Proposals of High-Frequency Noise Suppressions with Measured Data Studies

Appertaining to the results obtained in previous studies and based on the modeled data, it was found that the presence of the high-frequency noise affects the values of selected surface topography parameters, defined as ‘noise-sensitive parameters’ (NSP) [18], to a much greater extent than other parameters. The following parameters have been highlighted as an NSP: *Sz*, *Sdq*, *Sdr*, *Spd*, *Spc,* and *Sk*. It was also suggested to reduce the high-frequency noise with the biggest variations of values of the NSP and, simultaneously, with minimizing the differences in other (non-noise-sensitive) parameters.

Moreover, the results of noise removal, specified as an S-surface, can be studied to find the best solution from the considered commonly used algorithms. In general, the S-surface should both contain only those frequencies (visible in the PSD and ACF graphs) that are required to be removed from the raw measured data and maximizing the value of the peak density (*Spd*) parameter. In particular, the S-surface should contain the high-frequency components as its dominant frequency [63] or only frequencies in the high domain in the nominal, ideal case. Moreover, the S-surface should be isotropic in general, regardless of the type (direction) of the analyzed surface. Characterization of the process of noise removal can be provided with profiles (2D) and areal (3D) analysis.

**Figure 11:**
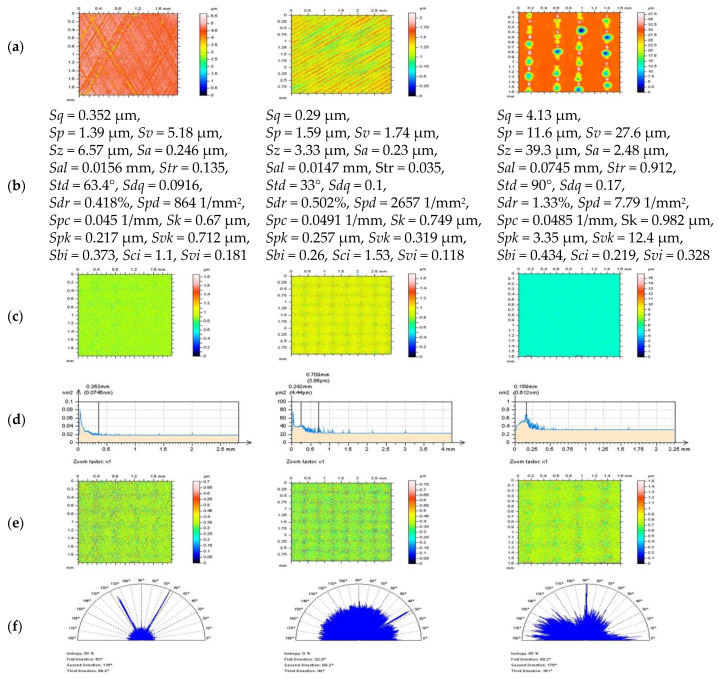
Defined for plateau-honed cylinder liner (left column), milled (center), and laser-textured (right column) surfaces: contour map plots of detail after DGS-F (cut-off = 0.025 mm) noise reduction (**a**) with selected surface topography parameters (**b**), correspondingly, received S-surface (**c**) with their PSDs (**d**), respectively, and S-surfaces after thresholding (0.13–99.87%) method (**e**) with their texture direction graphs (**f**) appropriately.

In Figure 11, results of removal of the high-frequency errors from plateau-honed cylinder liner, milled and laser-textured surfaces, by application of a regular Gaussian denoising filter, are presented. For improving the view validation of the algorithm, the thresholding method for S-surface processing was proposed. This technique was beneficial in recognition of the features located on the S-surface that are not in the high-frequency domain and, respectively, should not have been removed from the analyzed data (S-F surface). For plateau-honed surface, the S-surface contained the scratches, and when laser-textured topographies were studied, some laser treatment traces were located on the received noise surface as well. Gaussian denoising may be valuable for milled textures; nonetheless, the S-surface was not entirely in an isotropic manner. Reduction in sizes of the surface features, caused by an occurrence of features on the S-surface, is clearly undesirable in the processing of raw measured data.

**Figure 12:**
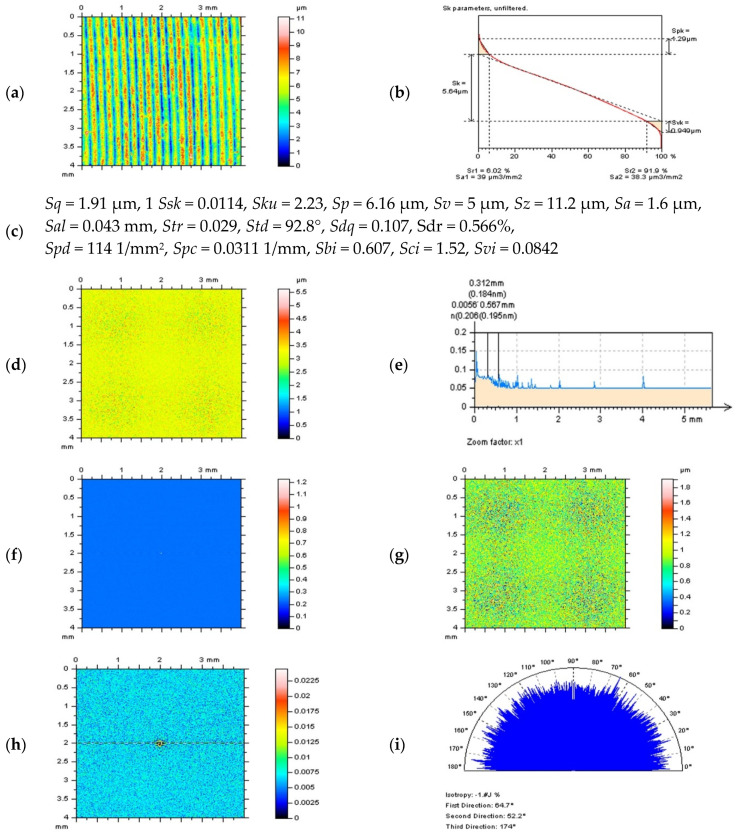
Contour map plot of the milled surface after a high-frequency errors reduction by a DMF method (**a**) with material ratio curve (**b**) and selected surface topography parameters (**c**) correspondingly, the S-surface received by application of DMF approach with cut-off = 0.025 mm (**d**), its PSD (**e**) and ACF (**f**), results of thresholding (0.13–99.87%) of S-surface (**g**) and ACF (**h**), the texture direction graph (**i**) defined for thresholded S-surface presented in (**g**) subsection of the figure.

In Figure 12 example of properly accomplished a denoising process was presented with a characterization (usage) of a regular median denoising filter. In this case, the thresholding technique was performed for both S-surface (g) and its ACF (h). In both instances, the S-surface did not contain surface finishing traces. The cut-off value also needs to be defined with analysis of an S-surface and its lack of manufacturing marks. When traces occur, then filter bandwidth should be reduced to receive non-feature S-surface. While DMF was applied, the cut-off was proposed with a 0.025 mm value. Moreover, the S-surface is also isotropic (i).

## 4. Conclusions

Characterization of surface topography, especially of engineering surfaces, is both a crucial matter and fraught with plenty of errors that can arise when measured data is processed. Despite this, some of the suggestions can arise:

1. Commonly used, available in the commercial software, algorithms and procedures, e.g., Power Spectral Densities (PSD), Autocorrelation Function (ACF), or filters such as ISO preferred Gaussian regression or robust filter, moving average (arithmetic mean), median or wavelet can be valuable in the definition of the S-F surface. Both operations, F-filtering and S-filtering, can be provided with regular, commercial procedures without alternative, external methods; nevertheless, special attention to the results obtained should be paid;
2. For defining the process of an areal form removal, particular attention must be paid to the minimization of a distortion of a selected surface topography features. Usually, distortion has a tendency to increase when deep or wide features, e.g., dimples, valleys, or scratches, were measured. Moreover, the density of the surface topography features affected the accuracy of an areal form removal (F operation) for each of the analyzed algorithms. Various methods, based on the exclusions (e.g., filling, extracting, omitting in general) of those relatively huge valleys, may be valuable in the minimization of errors in the computing of surface topography parameters;
3. Detection of the high-frequency errors from the results of surface topography measurements can be provided with an available in the commercial software Power Spectral Density or Autocorrelation Function graphs; nonetheless, both methods should be applied cautiously that they are vigilant against the occurrence of selected characteristics of the surface such as dimples, scratches, oil pockets, and valleys in general. The main purpose is to omit the deep and wide features when the PSD and ACF profile (or areal in exceptional cases) detection of high-frequency errors is accomplished. Other profile extraction methods, e.g., treatment traces or treatment direction methods (TDM), can also be valuable in the definition of this type of errors;
4. Reduction in the influence of the high-frequency noise on both data processing and parameter calculation errors can be minimized with an analysis of a noise surface (S-surface). It was found that S-surface, created in the process of removal of high-frequency errors from the raw surface topography measured data, should (must) be characterized by high-frequency components (at least high-frequency should be a dominant frequency with the PSD graph assessments), should not contain any manufacturing traces and be isotropic;
5. In general, all of the commercial algorithms (functions, filters) can be fairly advantageous in the characterization of the S-F surface (surface after removal of form and reduction in the high-frequency measurement errors) for various types of surface textures; nonetheless, application of algorithms must be sufficiently accurate, proposed procedures (valley extractions, thresholding approach or comprehensive analysis of the S-surface) may provide the more effective and detailed implementation of the widely available methods. Application of the external (other procedures than those available in the commercial software) may not be required when various modifications of the commonly used approaches, such as those proposed in this paper, will be approved. All the beneficial results obtained with external methods can be received with commercial procedures altered and supported by proposed schemes. Moreover, the extra time and researcher skills for the implementation of an external procedure will not be required.

In the future, the results of end-effect reduction in a digital filtration (not only those commonly used, available in commercial software) of different types of surface textures (excluding presented plateau-honed cylindrical topographies with additionally burnished dimples) will be published by the author.

## Figures and Tables

**Table 1:** Surface topography parameters calculated for plateau-honed cylinder liner surfaces with different densities (numbers) of oil pockets and after form removal by various techniques.

Parameters	Surf1				Surf2				Surf3			
	Poly 2nd	Poly 4th	Gauss 0.8 mm	Robust 0.8 mm	Poly 2nd	Poly 4th	Gauss 0.8 mm	Robust 0.8 mm	Poly 2nd	Poly 4th	Gauss 0.8 mm	Robust 0.8 mm
*Sq*, µm	1.75	1.72	1.69	1.83	1.97	1.91	1.94	2.01	2.15	2.12	2.09	2.19
*Ssk*	−2.64	2.56	−2.73	−2.71	−2.62	−2.39	−2.57	−2.54	−2.25	−2.15	−2.23	−2.29
*Sku*	10.6	10.5	11.4	10.9,	10.1	9.24	9.95	9.74,	7.82	7.51	7.9	7.85
*Sp*, µm	2.16	2.2	2.32	2.62	2.09	2.62	2.35	2.37	2.76	3.05	3.03	2.75
*Sv*, µm	9.54	9.28	9.42	9.86	9.56	10.1	9.43	9.67	9.5	9.31	9.39	9.61
*Sz*, µm	11.7	11.5	11.7	12.5	11.7	12.7	11.8	12	12.3	12.4	12.4	12.4
*Sa*, µm	1.13	1.12	1.07	1.16	1.31	1.28	1.27	1.33	1.47	1.46	1.4	1.5
*Sal*, mm	0.0615	0.0576	0.0599	0.0718	0.0707	0.0654	0.0705	0.0734	0.0675	0.064	0.0649	0.0701
*Str*	0.638	0.639	0.652	0.612	0.849	0.837	0.865	0.864	0.828	0.803	0.823	0.841
*Std*, °	63.7	63.7	63.6	63.7	117	117	117	117	117	117	116	117
*Sdq*	0.162	0.162	0.123	0.162	0.163	0.163	0.127	0.163	0.167	0.167	0.128	0.167
*Sdr*, %	1.24	1.24	0.735	1.24	1.28	1.28	0.787	1.28	1.32%	1.32	0.796	1.32
*Spd*, 1/mm^2^	159	164	44.9	122	172	122	45.9	154	132	108	32	126
*Spc*, 1/mm	0.0928	0.0922	0.0401	0.098	0.0906	0.0945	0.0381	0.0914	0.101	0.105	0.0383	0.102
*Sk*, µm	1.52	1.84	1.5	1.25	1.05	1.92	1.35	1.39	1.84	2.22	1.96	1.2
*Spk*, µm	0.243	0.232	0.398	0.521	0.22	0.391	0.537	0.439	0.392	0.318	0.643	0.555
*Svk*, µm	4.51	4.43	4.64	4.96	4.89	4.7	4.85	5.04	5.83	5.34	5.72	6.09
*Sbi*	2.1	2.15	1.73	1.57	2.54	1.92	2.53	2.52	2.02	1.69	1.8	2.1
*Sci*	0.489	0.555	0.521	0.511	0.403	0.6	0.563	0.527	0.516	0.586	0.619	0.495
*Svi*	0.276	0.263	0.277	0.289	0.263	0.254	0.26	0.261	0.276	0.265	0.277	0.287

**Table 2:** Surface topography parameters calculated for cylindrical turned or isotropic textures with different distributions of oil pockets and after form removal by various regular procedures.

Parameters	Surf1				Surf2				Surf3			
	Poly 2nd	Poly 4th	Gauss 0.8 mm	Robust 0.8 mm	Poly 2nd	Poly 4th	Gauss 0.8 mm	Robust 0.8 mm	Poly 2nd	Poly 4th	Gauss 0.8 mm	Robust 0.8 mm
*Sq*, µm	12.30	11.00	3.61	12.50	14.20	13.60	5.12	13.60	11.80	11.40	4.20	14.30
*Ssk*	−1.79	−1.43	1.02	−0.907	−1.25	−1.18	0.196	−1.21	−1.72	−1.57	−0.161	−1.74
*Sku*	5.13	4.86	6.78	6.06	3.52	3.34	3.98	3.98	5.47	5.24	3.62	6.10
*Sp*, µm	17.0	28.9	23.7	41.5	22.2	23.1	27.9	42.5	19.2	20.4	16.5	29.2
*Sv*, µm	43.40	41.30	11.30	52.70	39.60	38.10	9.74	41.90	41.20	42.30	9.75	61.80
*Sz*, µm	60.4	70.2	35.0	94.3	61.8	61.2	37.6	84.3	60.4	62.7	26.2	90.9
*Sa*, µm	8.87	8.00	2.37	7.48	11.10	11.00	3.79	10.30	8.35	8.15	3.01	9.58
*Sal*, mm	0.448	0.417	0.236	0.633	0.419	0.375	0.256	0.382	0.362	0.346	0.261	0.380
*Str*	0.859	0.885	0.126	0.315	0.951	0.950	0.550	0.720	0.977	0.976	0.556	0.777
*Std*, °	91.2	91.2	91.2	91.2	144.0	144.0	144.0	144.0	90.0	90.0	90.0	166.0
*Sdq*	0.0934	0.0945	0.0595	0.0962	0.6530	0.6540	0.3110	0.6540	0.7090	0.7090	0.324	0.709
*Sdr*, %	0.435	0.445	0.177	0.461	20.40	20.40	4.71	20.40	24.00	24.00	5.12	24.10
*Spd*, 1/mm^2^	1.180	0.623	2.180	0.187	212.00	207.00	233.00	72.800	265.00	227.00	437.00	57.50
*Spc*, 1/mm	0.0148	0.0153	0.0136	0.0294	0.417	0.417	0.188	0.477	0.415	0.423	0.17	0.493
*Sk*, µm	8.94	14.90	3.33	5.66	19.50	15.60	8.99	11.30	16.60	18.30	6.58	10.40
*Spk*, µm	3.72	6.64	6.74	21.20	2.47	3.03	6.01	10.60	2.73	2.92	5.49	12.10
*Svk*, µm	40.70	27.70	6.54	31.60	34.50	30.60	7.25	36.50	32.00	28.00	8.13	44.00
*Sbi*	1.870	0.642	0.206	0.577	1.900	1.400	0.255	0.495	1.600	1.430	0.445	1.180
*Sci*	0.548	0.837	1.620	1.370	0.841	0.800	1.410	0.915	0.743	0.858	1.500	0.893
*Svi*	0.310	0.251	0.163	0.236	0.199	0.186	0.152	0.202	0.265	0.242	0.190	0.309

**Table 3:** Surface texture parameters calculated for plateau-honed cylinder liners (left) and with additionally burnished dimples (right part) before and after noise, including and reduction by different regular filters.

Parameters	Cylinder Liner after A Plateau-Honing Process and after Noise Removal by Various Filters						Plateau-Honed Cylinder Liner with Additionally Burnished Dimples and after Noise Removal					
	Start Data	Noise Data	DGS-F	MADF	DMF	WDF	Start Data	Noise Data	DGS-F	MADF	DMF	WDF
*Sq*, µm	1.16	1.19	1.17	1.17	1.18	1.18	1.14	1.17	1.15	1.15	1.17	1.17
*Ssk*	0.535	0.532	0.550	0.555	0.545	0.552	−1.16	−1.11	−1.14	−1.15	−1.11	−1.11
*Sku*	2.34	2.42	2.36	2.34	2.39	2.37	5.93	5.94	6.06	6.07	5.96	5.93
*Sp*, µm	3.73	3.98	3.68	3.70	3.58	3.59	5.15	4.83	4.74	4.73	4.77	4.78
*Sv*, µm	6.05	6.35	5.84	5.65	5.84	5.74	5.30	5.93	5.71	5.69	5.71	5.68
*Sz*, µm	9.78	10.3	9.52	9.35	9.43	9.33	10.45	10.8	10.5	10.4	10.5	10.5
*Sa*, µm	0.997	1.000	0.994	0.993	0.999	0.997	0.850	0.827	0.818	0.816	0.826	0.826
*Sal*, mm	0.404	0.403	0.405	0.405	0.405	0.405	0.171	0.170	0.176	0.176	0.176	0.173
*Str*	0.390	0.389	0.388	0.388	0.391	0.391	0.213	0.217	0.225	0.225	0.225	0.221
*Std*, °	63.4	63.4	63.4	63.4	63.4	63.4	117	117	117	117	117	117
*Sdq*	0.0679	0.0982	0.0564	0.0490	0.0782	0.0577	0.1490	0.0898	0.0585	0.0542	0.0747	0.0623
*Sdr*, %	0.270	0.474	0.159	0.120	0.296	0.165	0.187	0.394	0.171	0.147	0.275	0.192
*Spd*, 1/mm^2^	35.3	185.0	22.7	14.7	56.5	8.99	10.7	40.0	11.3	10.5	16.3	5.05
*Spc*, 1/mm	0.1450	0.0579	0.0265	0.0231	0.0542	0.0207	0.0218	0.0656	0.0245	0.0230	0.0443	0.0161
*Sk*, µm	2.52	2.43	2.28	2.25	2.32	2.27	1.82	1.89	1.83	1.80	1.83	1.84
*Spk*, µm	1.45	1.62	1.69	1.70	1.70	1.72	1.07	1.03	1.03	1.04	1.06	1.04
*Svk*, µm	0.820	0.857	0.809	0.791	0.848	0.830	5.62	2.800	2.820	2.830	2.800	2.830
*Sbi*	0.843	0.674	0.792	0.778	0.867	0.858	0.371	0.375	0.378	0.378	0.381	0.380
*Sci*	1.84	1.83	1.84	1.84	1.84	1.84	1.29	1.30	1.29	1.29	1.30	1.30
*Svi*	0.0496	0.0532	0.0472	0.0457	0.0493	0.0471	0.185	0.183	0.183	0.182	0.183	0.184

**Table 4:** Surface texture parameters calculated for turned and ground surface before and after noise modeling and reduction by commonly used filters.

Parameters	Turned Surface Topography and after Noise Removal by Regular Filters						Ground Surface Topography and after A High-Frequency Noise-Removal Process					
	Start	Noise	DGS-F	MADF	DMF	WDF	Start	Noise	DGS-F	MADF	DMF	WDF
*Sq*, µm	1.89	1.98	1.89	1.88	1.9	1.88	0.332	0.351	0.320	0.314	0.330	0.322
*Ssk*	0.0130	0.0135	0.00819	0.00645	0.0106	0.00894	−0.690	−0.556	−0.644	−0.659	−0.626	−0.645
*Sku*	2.19	2.33	2.19	2.18	2.22	2.19	3.09	3.09	3.06	3.06	3.06	3.03
*Sp*, µm	5.57	7.29	5.94	5.92	6.15	5.97	1.050	1.570	0.970	0.869	1.010	0.882
*Sv*, µm	4.18	6.38	4.86	4.68	5.22	4.78	1.34	1.86	1.30	1.27	1.42	1.31
*Sz*, µm	9.75	13.7	10.8	10.6	11.4	10.8	2.39	3.43	2.27	2.14	2.44	2.19
*Sa*, µm	1.60	1.65	1.59	1.58	1.60	1.59	0.231	0.280	0.256	0.251	0.264	0.258
*Sal*, mm	0.043	0.043	0.043	0.043	0.043	0.043	0.0577	0.0551	0.0597	0.0608	0.0581	0.0597
*Str*	0.029	0.0301	0.029	0.029	0.029	0.029	0.0578	0.0497	0.0584	0.0584	0.0579	0.0584
*Std*, °	92.8	92.8	92.8	92.8	92.8	92.8	56.4	56.4	56.3	56.3	56.4	56.3
*Sdq*	0.0723	0.251	0.0862	0.0820	0.1300	0.0934	0.0445	0.0749	0.0413	0.0352	0.0565	0.0426
*Sdr*, %	0.261	3.1	0.371	0.336	0.845	0.435	0.5620	0.2800	0.0855	0.0619	0.1590	0.0909
*Spd*, 1/mm^2^	55.2	675.0	63.8	58.5	144.0	54.4	566	1531	873	632	1181	463
*Spc*, 1/mm	0.0116	0.1100	0.0195	0.0181	0.0584	0.0246	0.0166	0.0385	0.0162	0.0124	0.0318	0.0147
*Sk*, µm	5.6	5.84	5.58	5.55	5.63	5.59	0.750	0.850	0.766	0.754	0.792	0.774
*Spk*, µm	1.26	1.38	1.25	1.23	1.27	1.23	0.160	0.200	0.155	0.147	0.163	0.152
*Svk*, µm	0.845	1.190	0.908	0.890	0.941	0.889	0.452	0.472	0.450	0.445	0.461	0.451
*Sbi*	0.734	0.481	0.638	0.636	0.605	0.627	0.689	0.329	0.606	0.716	0.595	0.734
*Sci*	1.52	1.52	1.51	1.51	1.52	1.51	1.20	1.29	1.24	1.22	1.25	1.23
*Svi*	0.0822	0.0902	0.0831	0.0825	0.0841	0.0824	0.153	0.146	0.152	0.154	0.151	0.152

**Table 5:** Surface texture parameters calculated for milled (left part) and laser-textured (right part) surface before and after noise adding and reduction by different filters.

Parameters	Milled Surface Topography and after Noise Removal by Various Filters						Laser-Textured Surface Topography and after Noise Removal					
	Start Data	Data with Noise	DGS-F	MADF	DMF	WDF	Start Data	Data with Noise	DGS-F	MADF	DMF	WDF
*Sq*, µm	0.202	0.290	0.221	0.208	0.240	0.217	4.25	4.14	4.11	4.10	4.13	4.14
*Ssk*	−0.193	−0.0827	−0.119	−0.125	−0.116	−0.152	−2.50	−2.51	−2.52	−2.52	−2.51	−2.52
*Sku*	3.09	3.17	3.11	3.10	3.13	3.15	10.7	10.7	10.8	10.8	10.8	10.7
*Sp*, µm	0.864	1.59	0.891	0.780	0.999	0.855	12.7	13.4	12.8	12.6	12.6	12.4
*Sv*, µm	1.26	1.74	1.24	1.16	1.34	1.3	27.7	28.1	27.9	27.9	27.9	27.9
*Sz*, µm	2.124	3.330	2.130	1.940	2.340	2.150	40.4	41.5	40.7	40.4	40.6	40.3
*Sa*, µm	0.159	0.230	0.176	0.166	0.191	0.173	2.50	2.53	2.51	2.51	2.52	2.54
*Sal*, mm	0.0223	0.0147	0.0207	0.0211	0.0197	0.0207	0.0782	0.0784	0.0784	0.0784	0.0784	0.0784
*Str*	0.0220	0.035	0.0249	0.0231	0.0267	0.0241	0.876	0.878	0.877	0.875	0.877	0.871
*Std*, °	33	33	33	33	33	33	89.9	89.9	89.9	89.9	89.9	89.9
*Sdq*	0.0492	0.100	0.0500	0.0402	0.0715	0.0487	0.164	0.177	0.141	0.138	0.162	0.153
*Sdr*, %	0.1270	0.5020	0.1250	0.0808	0.2550	0.1180	0.969	1.38	0.963	0.928	1.190	1.050
*Spd*, 1/mm^2^	780	2657	1620	1289	2042	899	8.55	8.44	8.33	8.22	8.44	4.22
*Spc*, 1/mm	0.0349	0.0491	0.0203	0.0152	0.0407	0.0189	0.0322	0.0826	0.0332	0.0333	0.0601	0.0214
*Sk*, µm	0.603	0.749	0.581	0.546	0.626	0.564	4.09	4.02	4.07	4.04	4.06	4.03
*Spk*, µm	0.206	0.257	0.186	0.171	0.203	0.178	4.9	3.9	4.32	4.25	4.24	4.18
*Svk*, µm	0.236	0.319	0.239	0.228	0.259	0.244	12.6	13.2	13.1	13.1	13.3	13.4
*Sbi*	0.387	0.260	0.418	0.474	0.396	0.434	0.387	0.004	0.420	0.427	0.429	0.440
*Sci*	1.49	1.53	1.54	1.54	1.53	1.53	0.464	0.483	0.470	0.470	0.469	0.464
*Svi*	0.116	0.118	0.117	0.116	0.117	0.118	0.275	0.276	0.276	0.276	0.276	0.278

## Data Availability

Data sharing is not applicable to this article.

## Parameters and Abbreviations

The following abbreviations (left column) and parameters (right column) are used in the manuscript:

*ACF* autocorrelation function	*Sa* arithmetic mean height Sa, µm
*DGS-F* denoising Gaussian S-filter	*Sal* autocorrelation length, mm
*DMAF* denoising moving average filter	*Sbi* surface bearing index
*DMF* denoising median filter	*Sci* core fluid retention index
*DWF* wavelet denoising filter	*Sdq* root mean square gradient
*F-surface* form surface	*Sdr* developed interfacial areal ratio, %
*Gauss* regular Gaussian regression filter	*Sk* core roughness depth, µm
*MQCL* Minimum Quantity Cooling Lubrication	*Sku* kurtosis
*NSP* noise-sensitive parameters	*Sp* maximum peak height, µm
*Poly 2nd* polynomial of 2nd order	*Spc* arithmetic mean peak curvature, 1/mm
*Poly 4th* polynomial of 4th order	*Spd* peak density, 1/mm^2^
*PSD* power spectral density	*Spk* reduced summit height, µm
*Robust* regular robust Gaussian regression filter	*Sq* root mean square height, µm
*S-F surface* A surface after S- and F- filtering	*Ssk* skewness
*S-filter* removes small-scale lateral components	*Std* texture direction,°
*S-surface* short-wavelength noise surface	*Str* texture parameter
*SLM* selective laser melting	*Sv* maximum valley depth, µm
*ST* surface topography	*Svi* valley fluid retention index
*TDM* treatment-(traces)-direction-method	*Svk* reduced valley depth, µm
*VEM* valley excluding method	*Sz* the maximum height of surface, µm
*WLI* White Light Interference	
